# Family medicine practitioners’ stress during the COVID-19 pandemic: a cross-sectional survey

**DOI:** 10.1186/s12875-021-01382-3

**Published:** 2021-02-14

**Authors:** Marion Dutour, Anna Kirchhoff, Cécile Janssen, Sabine Meleze, Hélène Chevalier, Sandrine Levy-Amon, Marc-Antoine Detrez, Emilie Piet, Tristan Delory

**Affiliations:** 1 Infectious disease department, Annecy-Genevois hospital, Annecy, France; 2 Institut national d’études démographiques (Ined), UR 14, Aubervillers, France; 3 Helene Chevalier Conseils, Paris, France; 4 Stimulus, Paris, France; 5 Clinical research unit, Annecy-Genevois hospital, Annecy, France

**Keywords:** COVID-19, Pandemics, Stress, General practitioners, Cross-sectional survey

## Abstract

**Background:**

The COVID-19 pandemic has shaken the world in early 2020. In France, General Practitioners (GPs) were not involved in the care organization’s decision-making process before and during the first wave of the COVID-19 pandemic. This omission could have generated stress for GPs. We aimed first to estimate the self-perception of stress as defined by the 10-item Perceived Stress Score (PSS-10), at the beginning of the pandemic in France, among GPs from the Auvergne-Rhône-Alpes, a french administrative area severely impacted by COVID-19. Second, we aimed to identify factors associated with a self-perceived stress (PSS-10 ≥ 27) among socio-demographic characteristics of GPs, their access to reliable information and to personal protective equipment during the pandemic, and their exposure to well established psychosocial risk at work.

**Methods:**

We conducted an online cross-sectional survey between 8th April and 10th May 2020. The self-perception of stress was evaluated using the PSS-10, so to see the proportion of “not stressed” (≤20), “borderline” (21 ≤ PSS-10 ≤ 26), and “stressed” (≥27) GPs. The agreement to 31 positive assertions related to possible sources of stress identified by the scientific study committee was measured using a 10-point numeric scale. In complete cases, factors associated with stress (PSS-10 ≥ 27) were investigated using logistic regression, adjusted on gender, age and practice location. A supplementary analysis of the *verbatims* was made.

**Results:**

Overall, 898 individual answers were collected, of which 879 were complete. A total of 437 GPs (49%) were stressed (PSS-10 ≥ 27), and 283 GPs (32%) had a very high level of stress (PSS-10 ≥ 30). Self-perceived stress was associated with multiple components, and involved classic psychosocial risk factors such as emotional requirements. However, in this context of health crisis, the primary source of stress was the diversity and quantity of information from diverse sources (614 GPs (69%, OR = 2.21, 95%CI [1.40–3.50], *p* < 0.001). Analysis of *verbatims* revealed that GPs felt isolated in a hospital-based model.

**Conclusion:**

The first wave of the pandemic was a source of stress for GPs. The diversity and quantity of information received from the health authorities were among the main sources of stress.

**Supplementary Information:**

The online version contains supplementary material available at 10.1186/s12875-021-01382-3.

## Background

In December 2019, the COVID-19 (SARS-COV-2 virus) epidemic started in Wuhan, China [1]. The pandemic state was declared on 11th of March 2020 by the World Health Organization (WHO), and its impact on every part of daily life means the COVID-19 pandemic is one of the most significant events of the twenty-first century.

In France, by August 2020, ~ 100,000 severe cases had been hospitalized and ~ 30,000 deaths had occurred. To tackle the epidemic, the French government applied a strategy which was predefined after the 2009 H1N1 epidemic, and inspired by the Chinese and Italian management of COVID-19 [2]. Several phases were organized, thanks to the Operational Coordination of Epidemic and Biological Risks (COREB), which promoted exchanges between numerous specialists, though excluding General Practitioners (GPs) from the decision process [3].

The first phase aimed to slow down the introduction of the SARS-COV-2 virus on the French territory. The second one consisted of preventing the virus from spreading throughout the country. To do this, it was decided that any patient suspected of COVID-19 would have to be admitted to referral hospitals [4]. On 14th March 2020, 4500 patients had been diagnosed with COVID-19, the critical care units of eastern and northern France, and the Paris area, were being overwhelmed when the French government initiated a national lockdown [5, 6]. In addition to the lockdown, a decision was made to switch the management of mild cases from hospitals to primary care in family practices [7]. During this phase, GPs officially became part of the pandemic response organization and had to adapt their practices and workflow urgently. At the same time, shortage of personal protective equipment (PPE) happened worldwide [8].

The unique and unpredictable nature of this pandemic urged the WHO to warn about the possible occurrence of professional stress and psychological disorders [9].

First, our hypothesis was that due to a lack of initial involvement of GPs in the crisis management and decision-making during the first wave of the pandemic, they might have suffered from professional stress disorders. We also hypothesized that socio-demographic characteristics of GPs, their access to reliable information and to personal protection equipment (PPE) may have mitigated their self-perception of stress.

We first aimed to estimate the self-perception of stress, at the beginning of the pandemic in France, as measured by the 10-item Perceived Stress Score (PSS-10), among GPs from the Auvergne-Rhône-Alpes, a French administrative area severely impacted by COVID-19, so to see the proportion of “not stressed”, “borderline”, and “stressed” GPs. Then we aimed to identify factors associated with a self-perceived stress (PSS-10 ≥ 27), among socio-demographic characteristics of GPs, their access to reliable information and to PPE during the pandemic, and their exposure to well established psychosocial risk at work.

## Methods

### Study design and participants

We conducted an online cross-sectional survey of every GP registered in the Auvergne-Rhône-Alpes region, a 7,948,287 inhabitant’s administrative area where the first clusters of cases were reported [10]. The eligibility criteria was to be a GP registered in the Auvergne-Rhône-Alpes area, agreeing for data collection and analysis. Exclusion criteria was to be a medical student or a GP working exclusively in hospitals. To reach the GPs, we released our survey through the official mailing list of medical professionals’ network for the area (union régionale des professionnels de santé, URPS). This mailing list included 5344 GPs. They were invited to fill an online self-questionnaire, through the software of a private society (GIDE). This cross-sectional survey was conducted from the perspective of a prospective cohort to monitor the self-perceived stress of GPs of the Auvergne-Rhône-Alpes area over the first wave of the pandemic. Each GP participating in the survey were asked to be enrolled within the cohort. Herein we are reporting the results of this baseline assessment, composed of answers collected from 8th April to 10th May 2020. Throughout the study period, ~ 10% of French COVID-19 cases and deaths occurred in this area, and the national lockdown was in place.

### Measurements

The self-questionnaire was regrouping five main parts: 1) socio-demographic characteristics of GPs, 2) practice and workflow before and during the pandemic, 3) their self-perception of stress according to the PSS-10 validated in French, 4) their agreement with 31 positive assertions to identify potential origin of stress, and 5) an open-ended question (Supplementary Table 1). It was devised by the scientific study committee, involving: 2 residents in family medicine, 2 infectious diseases physicians, 3 specialists in epidemiological studies including prevention of stress at work, and 1 methodologist. It was tested on 12 GPs in a pilot phase to ensure the comprehension and calibration of the questions.

The Perceived Stress Score with 10 items (PSS-10) was used to assess the level of self-perceived stress [11]. It can be self-administered, and is validated for French with a Cronbach’ alpha coefficient above 0.70 [12]. The PSS-10 allocates GPs under three categories: “not stressed” (PSS-10 ≤ 20), “borderline” (21 ≤ PSS-10 ≤ 26), and “stressed” (PSS-10 ≥ 27) [11]. We also identified and analyzed a “very stressed” (PSS-10 ≥ 30) category [11].

The 31 positive assertions were developed by the scientific study committee to explore the root-causes of stress. They were regrouped under 8 themes: 1) workload (3 assertions); 2) emotional requirements (5 assertions); 3) conflict of values (5 assertions); 4) economic insecurity (2 assertions); 5) working relationships and social reports (4 assertions); 6) personal protection equipment (5 assertions); 7) access to information (4 assertions); and 8) others (3 assertions) [13]. For each affirmation, GPs had to express their agreement using a 10-point numeric scale, ranging from one (“I do not agree at all”) to ten (“I totally agree”). An agreement ≥6/10 was considered as “agree”, while an agreement < 6/10 was considered as “not agree”.

At last, an open-ended question was left to collect any remarks from the GPs. *Verbatims* of the open-ended question were planned to be analyzed to identify other possible sources of stress.

### Data, *verbatims* and statistical analysis

Data collected were analyzed by the clinical research unit of the Annecy hospital: a statistician for quantitative data and two GPs as independent reviewers for the *verbatims* analysis. To ensure that the sampling obtained through the mailing was representative of the GP population, we compared the sample’s demographic characteristics to the real demographic characteristics of GPs available at regional level from the URPS in 2020, and at the national level from the French Ministry of Health in 2018. In this cross-sectional study we use Student t-test, Pearson’s correlation test and Chi-squared test for univariate comparison.

In complete cases, we explored factors associated with a self-perceived stress defined as a PSS-10 ≥ 27, using a multivariate logistic regression adjusted for gender, age as continuous value, and practice location. No imputation of missing data was performed. The variable selection was made using a backward elimination based on the Akaike criterion. The associations are represented using odds ratio (OR) with their 95% confidence interval (95CI). The level of significance was set at 5% bilateral. Statistical analysis was performed using the R software version.4.0.2. (the R foundation, Vienna, Austria).

The *verbatims* of the open-ended question were handled as qualitative data. They were double-read by two independent reviewers. The reviewers extracted and categorized the answers under the 8 themes identified to develop the 31 positive assertions. Whenever the two reviewers could not classify GPs’ answers under existing themes, they confronted their extraction to categorize them under new themes they defined together.

## Results

### General practitioner population

Over the study period, we collected 1050 GPs’ responses, of whom 5 worked exclusively in the hospital, 6 were not actually in the targeted administrative area, and 141 (13%) have not validated their questionnaire. Therefore, the final set used for analysis was constituted of 898 GPs’ responses (17% of the 5344 emails sent, and 86% of the initial responses), of which 879 were complete.

Table 1 describes GPs’ characteristics. The majority of respondents were women (60.5%) and the average age was 47.7 years. Women were younger than men (44.9 vs. 51.9 years, *p* < 0.001). The sample was representative of the GP population in the area, with a small gap in gender (61% of women vs. 51 to 54% in the control data from URPS and the French Ministry of Health) and age (47 on average vs. 48 to 52 in control data).

**Table 1:** General Practitioners’ characteristics

Characteristics	Overall *N* = 898		Men *N* = 355 (39.5%)		Women *N* = 543 (60.5%)		*P*-value
	N / Mean	(%) / SE	N / Mean	(%) / SE	N / Mean	(%) / SE	
Age	47.7	± 11.4	51.9	± 11.4	44.9	± 10.6	< 0.001
Years of practice	14.9	± 11.5	19.3	± 12.2	12.1	±10.1	< 0.001
Number of patients per week before the pandemic	95	± 37	110	± 41	85	± 30	< 0.001
Exercise localization							
Rural	146	(16%)	68	(19%)	78	(14%)	0.040
Semi rural	349	(39%)	129	36%)	220	(41%)	0.200
Urbain	409	(46%)	158	(45%)	247	(46%)	0.700
Practice	898		355		543		< 0.001
Alone	222	(25%)	108	(31%)	111	(21%)	< 0.001
In an office, with other GPs	526	(59%)	183	(52%)	340	(63%)	< 0.001
In a multidisciplinary nursing home	156	(17%)	64	(18%)	92	(17%)	0.700

### Prevalence of self-perceived stress (PSS-10 score)

Overall, the average PSS-10 score was 26.4 (±6.4): 169 GPs (19%) were “not stressed” (PSS-10 ≤ 20), 292 (33%) “borderline” (21 ≤ PSS-10 ≤ 26), and 437 (49%) “stressed” (PSS-10 ≥ 27). A total of 283 GPs (32%) were “very stressed” (PSS-10 ≥ 30).

### Impact of the epidemic on the practice

They were 880 (98%) GPs to adapt their practice and facilities to implement barrier measures. Issues in obtaining protective equipment were reported by 531 (59%) of them. Most of GPs (741, 83%) considered having less work over the study period (Table 2).

**Table 2:** General Practitioners’ practice before and during the pandemic

	Before the pandemic	During the pandemic^ a^	*P*-value
Number of patients per week	95.1 ± 37.3	45.2 ± 28.7	< 0.001
Daily hours spent on the phone with patients	1.3 ± 1.1	2.1 ± 1.4	< 0.001
Use of teleconsultation, yes	126 (14%)	765 (86%)	< 0.001
Perform home-visits, yes	815 (91%)	646 (72%)	< 0.001

^a^ The answers are representative of the practices in the week before answering to the self-questionnaire

### Factors associated with stress

Two-third of GPs had someone to talk to (556, 62%), they were mostly supported by their family and friends (617, 69%), and very few had the willingness to withdraw from practice (46, 7%).

Table 3 details the results of the multivariate analysis of complete cases. Over 879 GPs, women were twice as stressed as men (OR = 1.88, 95%CI [1.31–2.72], *p* < 0.001). In addition to gender, other factors were found being a source of self-perceived stress among the 31 positive assertions: making difficult decisions, being affected by patient anxiety, being overwhelmed by information, having a heavy workload, having the feeling of being alone, and the feeling that work time impacted on personal life. Factors associated with lesser stress were: being in line with the job, having the feeling of being useful, having trust in the future and having the ability to forget work when reaching home.

**Table 3:** Factors associated to a self-perceived stress (PSS-10 ≥ 27) among GPs, multivariate analysis on complete cases (*n* = 879)

Adjustment variables ^**a**^	N 879 (100%)	PSS-10 ^**a**^ < 27 455 (51.8%)	PSS-10 ^**a**^ ≥ 27 424 (48.2%)	Adjusted OR	95CI	***P***-value
**Demographics**						
Age (per year increase), Mean (years) +/− SE	47.7 ± 11.4	48.8 ± 12.0	46.3 ± 10.7	0.99	[0.98 to 1.01]	0.400
Sex, women	532 (60.5%)	232 (26.4%)	300 (34.1%)	1.88	[1.31 to 2.72]	< 0.001
Exercise locations						
Rural	139 (15.8%)	82 (9.3%)	57 (6.5%)	Ref		
Semi-rural	340 (38.7%)	161 (18.3%)	179 (20.4%)	1.32	[0.76 to 2.28]	0.300
Urbain	400 (45.5%)	212 (24.1%)	188 (21.4%)	1.32	[0.77 to 2.29]	0.300
**Agreement to the positive assertions using a 10-point agreement score (AS)** ^**b**^						
**Workload**						
*I have an inordinate amount of work.*						
1 ≤ AS ≤5	726 (82.5%)	410 (46.6%)	316 (36.0%)	ref		
6 ≤ AS ≤8	124 (14.1%)	36 (4.1%)	88 (10.0%)	2.06	[1.18 to 3.64]	0.012
9 ≤ AS ≤10	29 (3.4%)	9 (1.0%)	20 (2.3%)	1.81	[0.69 to 4.97]	0.200
*I feel like my work is taking up so much of my time that it’s impacting my personal life*						
1 ≤ AS ≤5	559 (63.5%)	339 (38.6%)	220 (25.0%)	ref		
6 ≤ AS ≤8	199 (22.6%)	85 (9.7%)	114 (13.0%)	1.19	[0.77 to 1.82]	0.400
9 ≤ AS ≤10	121 (13.9%)	31 (3.5%)	90 (10.2%)	2.15	[1.16 to 4.06]	0.020
**Emotional requirements**						
*I am affected by my patients anxiety or anguish*						
1 ≤ AS ≤5	451 (51.3%)	317 (30.1%)	134 (15.2%)	ref		
6 ≤ AS ≤8	314 (35.7%)	113 (12.9%)	201 (22.9%)	2.37	[1.64 to 3.44]	< 0.001
9 ≤ AS ≤10	114 (13.0%)	25 (2.8%)	89 (10.1%)	3.41	[1.87 to 6.36]	< 0.001
*When I get home, I can forget about my work*						
1 ≤ AS ≤5	468 (53.2%)	176 (20.0%)	292 (33.2%)	ref		
6 ≤ AS ≤8	244 (27.8%)	150 (17.1%)	94 (10.7%)	0.5	[0.34 to 0.74]	0.003
9 ≤ AS ≤10	167 (19.0%)	129 (14.7%)	38 (4.3%)	0.3	[0.19 to 0.51]	< 0.001
**Conflict of values**						
*I feel in line with what I do in my job*						
1 ≤ AS ≤5	197 (22.4%)	56 (6.4%)	141 (16.0%)	ref		
6 ≤ AS ≤8	394 (44.8%)	197 (22.4%)	197 (22.4%)	0.56	[0.35 to 0.89]	0.020
9 ≤ AS ≤10	298 (32.8%)	202 (23.0%)	86 (9.8%)	0.3	[0.18 to 0.50]	< 0.001
*My work is useful to the community*						
1 ≤ AS ≤5	130 (14.8%)	44 (5.0%)	86 (9.8%)	ref		
6 ≤ AS ≤8	336 (38.2%)	171 (19.4%)	165 (18.8%)	0.55	[0.32 to 0.94]	0.030
9 ≤ AS ≤10	412 (47.0%)	240 (27.3%)	173 (19.7%)	0.54	[0.31 to 0.93]	0.030
**Economic insecurity**						
*I am confident in the future / able to project myself in the coming weeks*						
1 ≤ AS ≤5	333 (50.2%)	160 (18.2%)	281 (32.0%)	ref		
6 ≤ AS ≤8	268 (30.5%)	158 (18.0%)	110 (12.5%)	0.64	[0.43 to 0.95]	0.020
9 ≤ AS ≤10	170 (19.3%)	137 (15.6%)	33 (3.7%)	0.31	[0.18 to 0.51]	< 0.001
**Working relationship and social reports**						
*I feel lonely in my work*						
1 ≤ AS ≤5	633 (72.0%)	369 (42.0%)	264 (30.0%)	ref		
6 ≤ AS ≤8	150 (16.1%)	60 (6.8%)	90 (10.2%)	1.56	[0.98 to 2.47]	0.060
9 ≤ AS ≤10	96 (10.9%)	26 (3.0%)	70 (8.0%)	2.18	[1.18 to 4.11]	0.010
**Access to information**						
*I feel overwhelmed by the amount and variety of information I receive*						
1 ≤ AS ≤5	277 (31.5%)	192 (21.8%)	85 (9.7%)	ref		
6 ≤ AS ≤8	335 (38.2%)	168 (19.1%)	167 (19.0%)	1.4	[0.92 to 2.13]	0.100
9 ≤ AS ≤10	267 (29.3%)	95 (10.8%)	172 (19.6%)	2.21	[1.40 to 3.50]	< 0.001
**Others**						
*I have some tough decisions to make*						
1 ≤ AS ≤5	503 (57.2%)	321 (36.5%)	182 (20.7%)	ref		
6 ≤ AS ≤8	276 (31.4%)	106 (12.1%)	170 (19.3%)	2.07	[1.41 to 3.06]	< 0.001
9 ≤ AS ≤10	100 (11.4%)	28 (3.2%)	72 (8.2%)	2.45	[1.35 to 4.51]	0.003

^a^ Of the 31 positive assertions, only 10 remained in the multivariable model after backward selection

^b^ The 10-point agreement score (AS) is based on a numeric scale ranging from one « I do not agree at all » to ten « I totally agree ». An agreement score ≥ 6/10 was considered as “agree”, while an agreement < 6/10 was considered as “not agree”

### Analysis of *verbatims*

We collected 294 answers through the last open-ended question, of which 202 were suitable for *verbatims* analysis (Supplementary Table 2). A total of 153 *verbatims* were related to the 8 themes developed for the 31 positive assertions, and 173 *verbatims* were about 5 new themes. The three main new themes were: 1) The place of GPs in the organization of the response to the health crisis (*n* = 72); 2) The feeling of GPs during health crisis (*n* = 67); and 3) New concepts about information and guidelines (*n* = 36). From these three new themes we could describe three networks for communication around GPs during the health crisis. The first one was between health authorities and GPs. In this first network, the communication was a one-way flow from health authorities to GPs. GPs considered that information they received was too vague, unsuitable to their practice, and sometimes contradictory (*n* = 58). They described a difficulty to assimilate knowledge as guidelines were frequently modified (*n* = 29). Few noted that they had the same type and level of information as their patients (*n* = 8). The second network was a two-way flow between hospitals and GPs, which was sometimes reported as being non-existent (*n* = 13). This lack of cooperation has led to feelings of frustration and sometimes even guilt (*n* = 7). The last network was a two-way flow between GPs themselves, and was reported. It has been in many places as being a source of mutual help (*n* = 29). Finally, GPs had the feeling of being neglected, not taken seriously and not fully considered by health authorities (*n* = 49), while they were standing alone in the hospital-based response to the pandemic (*n* = 43).

## Discussion

Our survey reveals that the level of stress was very high in GPs during the first wave of the COVID-19 pandemic within lockdown phase in France, as half of the GPs were stressed (PSS-10 ≥ 27), and a third very stressed (PSS-10 ≥ 30).

A Danish study conducted on 3350 GPs in a non-pandemic period, also using PSS-10, showed that the baseline level of stress can be as high as 21% of GPs, much lower than the 49% observed in our study [14]. Though French and Danish GPs are likely to be not comparable, it is estimated that in Europe, 25% of workers present stress related to work, which is also much lower than the rate we observed [15, 16]. This significantly different rate of stress is likely to be due to the pandemic’s context and the lockdown, though no baseline evaluation of stress in GPs was performed prior to these events. The pandemic was reported as a source of stress in the general population and among hospital medical staff, and there is no reason to consider that GPs were spared [17–19].

Besides this stressful context, we identified independent sources of stress. Contrary to popular belief, one of these sources was not the workload, but rather the change in practice. In addition to the reorganization and redesign of facilities, the workload of GPs decreased significantly from 95.1 to 45.2 patients per week. This decrease was confirmed by results from a flash survey conducted by the French government in April 2020 which showed a diminution of consultations for patients with underlying and chronic condition, pediatric and pregnancy follow [20]. On the contrary, an increase in consultations related to psychological distress was observed over the same period [20]. Similar observations were made in England, consultation design was shown to be modified, switching from face-to-face to remote consultations [21]. Those observations corroborated our results about the number of GPs using teleconsultation during the pandemic, from 14% before to 86% during the pandemic. However, neither of these studies, nor ours, analysed a potential link between the change in consultation profile and the subsequent workload, and the occurrence of a self-perceived stress among GPs.

Previous studies showed that GPs aren’t sufficiently trained and prepared for health crises [22, 23]. Although there were predefined plans to respond to a pandemic, we believe it is important to highlight that there was a lack of collaboration between GPs and the health care authorities [24]. It was not limited to the French context, and it appears to be a root-cause of professional stress [24]. In addition, in a context of shortage of protective equipment, GPs had to modify their practices quickly and constantly adapt their local organization as they received multiple conflicting and late information from the health authorities.

GPs suffered from a health policy considered as too hospital-based. This pre-existing lack of communication and collaboration between GPs and hospitals was revealed by the pandemic [25]. To assist GPs and minimize their likelihood of stress in a health crisis, it might be beneficial to improve their level of information. Health authorities and the media are playing a crucial role in crisis situations and effective communication strategies have shown to improve the dissemination of accurate and appropriate information [26, 27]. As highlighted by Desborough J et al., our results urge that a single source of reliable information from health authorities is needed in time of crisis, both for clinicians and the public [28].

As we conducted our study in real-time, GPs were questioned at the heart of the first pandemic wave and during the lockdown in France. This timing means that GPs answers might have been more spontaneous and closer to their feelings about the health crisis. It also biased the analysis of the determinant of stress, as no baseline evaluation was available. In France, women are more representative of the young generation of GPs [29]. As the census was held online, the young generation was likely to be prompt for answer [30]. It could explain the biased selection of women in our study. Women appeared more stressed, which could increase the mean PSS-10 score. But this trend is well known and described for the PSS-10 score [31]. We used the PSS-10 score, as it has the advantage of being short and quick to fill, while it provides a quantitative measurement of the subjective dimension of stress. Some studies prefer other tools, often associated, to explore different dimensions of well-being, of which we inspired to construct our agreement items [32, 33]. In this particular context, some stress scales have been created to better understand and specifically assess COVID-19-related distress [34]. But those stress scales were validated after the beginning of our study [34]. In our study, while quantitative analysis revealed several causes of stress, many may remain unknown, as underlined by the analysis of *verbatims*. Qualitative studies are needed to address this issue.

## Conclusions

The COVID-19 pandemic was very stressful for GPs at the time of the first wave, during the lockdown. A structured and non-controversial chain of communication from the health authorities is important to ensure GP’s confidence. The pandemic underlines the importance of GPs and the liberal network in a health crisis, to provide an ambulatory follow-up of patients with continuity of care. It is therefore crucial to fully integrate GPs in the management and decision process of a health crisis. Links with local hospitals should be developed. A prospective follow-up of GP’s stress during the different phases of the epidemic is needed to strengthen the identification of stress determinants and their evolution.

## Supplementary Information

**Additional file 1: Table S1.** English version of the self-questionnaire.

**Additional file 2: Table S2.** Topics classification of the open-ended question of the self-questionnaire.

## Data Availability

The datasets used to conduct the analysis are not deposited in a public repository. They can be made available to academic researchers upon request to the study scientific committee. Requests have to be addressed to the corresponding author.

## Abbreviations

AS: Agreement Score
GP: General Practitioners
OR: Odds Ratio
COREB: Operational Coordination of Epidemic and Biological Risks
PSS: Perceived Stress Score
URPS: Union Régionale des Professionnels de Santé
WHO: World Health Organization
