# Supplementary material for: Family medicine practitioners’ stress during the COVID-19 pandemic: a cross-sectional survey
Source: BMC Fam Pract. 2021 Feb 14;22:36. doi: 10.1186/s12875-021-01382-3 (PMC7882249; doi:10.1186/s12875-021-01382-3)
Supplement: Supplementary file 1 — Additional file 1: Table S1. English version of the self-questionnaire. [file 12875_2021_1382_MOESM1_ESM.docx]

**Supplementary Table 1. English version of the self-questionnaire.**

| **GPs stress during the COVID-19 pandemic**  In the context of COVID 19, we are proposing a survey to you, general practitioners, to study your experiences during this crisis period.  The duration of this questionnaire is estimated at 10 minutes.  In order to measure the evolution of your feelings during the pandemic, we would like to repeat this same questionnaire at three forthcoming time-points over the pandemic.  If you wish to be part of this sentinel group, please let us known your email address: ....  The results will be communicated to you at the end of the study.  All answers are treated in compliance with the GDPR (General Data Protection Regulation). | | | | | | | | |
| --- | --- | --- | --- | --- | --- | --- | --- | --- |
| This study aims to enroll experienced general practitioners working in ambulatory care.  Thus, if you practice exclusively in hospitals, this study is not intended for you. | | | | | | | | |
| 0 | Do you currently work exclusively at the hospital? | Yes  No | | | | | | |
| **To begin, a few questions about your profile:** | | | | | | | | |
| 1 | Age | _ _ (years) | | | | | | |
| 2 | Sex | Man  Woman | | | | | | |
| 3 | You are… | Established  Substitute  Medical student | | | | | | |
| 4 | How many years have you been in this medical office? | _ _ (years) | | | | | | |
| 4bis | In which department do you work? | _ _ (number from 01 to 95) | | | | | | |
| 5 | How far away is the nearest hospital? | _ _ (in minutes, by car, in a normal conditions) | | | | | | |
| 6 | Where do you practice? | In rural area  In semi-rural area  In urban area | | | | | | |
| **About your exercise:**  Do you practice… | | | | | | | | |
| 7 | … in ambulatory? | Alone  In an office, with other GPs  In a multidisciplinary nursing home | | | | | | |
| 8 | … in hospital? | Yes  No  If yes, number of half-days per week: _ _  Or per month: _ _ (if <1/week) | | | | | | |
| 9 | ... in Senior care facility, as coordinating physician? | Yes  No  If yes, number of half-days per week: _ _  Or per month: _ _ (if <1/week) | | | | | | |
| 10 | ... in an emergency medical service control center? | Yes  No  If yes, number of days/on call per week: _ _  Or per month: _ _ (if <1/week) | | | | | | |
| 11 | Do you have a specific activity (pediatrics, gynecology, mountain medicine, homeopathy...)? | Yes, please specify : …  No | | | | | | |
| 12 | Do you vaccinate against the flu? | Yes every year  Yes sometime  No | | | | | | |
| **About how you have felt in the last four weeks:**  The questions in this scale ask you about ***your feelings* and *thoughts* during the last month**.  Although some of the questions are similar, there are differences between them, and you should treat each one as a separate question. In each question, you will be asked to indicate your response representing HOW OFTEN you felt or thought a certain way.  The best approach is to answer fairly quickly. That is, don’t try to count up the number of times you felt a particular way, but rather indicate the alternative that seems like a reasonable estimate. | | | | | | | | |
|  | In the last month, how often… | Never | | Almost never | | Sometimes | Fairly often | Very often |
| 13 | … have you been upset because of something that happened unexpectedly? | 1 | | 2 | | 3 | 4 | 5 |
| 14 | … have you felt that you were unable to control the important things in your life? | 1 | | 2 | | 3 | 4 | 5 |
| 15 | … have you felt nervous and “stressed”? | 1 | | 2 | | 3 | 4 | 5 |
| 16 | … have you felt confident about your ability to handle your personal problems? | 5 | | 4 | | 3 | 2 | 1 |
| 17 | … have you felt that things were going your way? | 5 | | 4 | | 3 | 2 | 1 |
| 18 | … have you found that you could not cope with all the things that you had to do? | 1 | | 2 | | 3 | 4 | 5 |
| 19 | … have you been able to control irritations in your life? | 5 | | 4 | | 3 | 2 | 1 |
| 20 | … have you felt that you were on top of things? | 5 | | 4 | | 3 | 2 | 1 |
| 21 | … have you been angered because of things that were outside of your control? | 1 | | 2 | | 3 | 4 | 5 |
| 22 | … have you felt difficulties were piling up so high that you could not overcome them? | 1 | | 2 | | 3 | 4 | 5 |
| **About your usual practice, during an ordinary week, before the pandemic :** | | | | | | | | |
| 23 | Approximately how many patients do you see per week in consultations? | | _ _ _ | | | | | |
| 24 | How much time do you spend on the phone on average over a day with patients (excluding paid teleconsultations)? | | _ (in hours) | | | | | |
| 25 | Do you practice teleconsultation? | | Yes  If yes, about how much per week : _ _  No | | | | | |
| 26 | Approximately how many home visits do you make per week? | | _ _ | | | | | |
| **About your practice during this last week:** | | | | | | | | |
| 27 | Approximately how many patients do you see per week in consultations? | | _ _ _ | | | | | |
| 28 | How much time do you spend on the phone on average over a day with patients (excluding paid teleconsultations)? | | _ (in hours) | | | | | |
| 29 | Do you practice teleconsultation? | | Yes  If yes, about how much per week : _ _  No | | | | | |
| 30 | Approximately how many home visits do you make per week? | | _ _ | | | | | |
| 31 | In the past week, what was the proportion of suspected or confirmed cases in your patient? | | None  A minority  Half  The majority  All of it | | | | | |
| 32 | Did any of your patient died from COVID-19? | | Yes  No  If yes: How many (in total since the beginning of the epidemic)? _ _ _ | | | | | |
| **And today, how do you feel about COVID-19?** | | | | | | | | |
| 33 | I am infected or have been infected with Covid-19? | | Yes, I am  Yes, I was  No | | | | | |
| 34 | {If “No” to Q.33} I'm afraid I'm already infected without knowing it. | | Yes  No | | | | | |
| 35 | {If “No” to Q.33} I'm afraid I may be infected in the next few weeks | | Yes  No | | | | | |
| **On a scale of 1 to 10,**  **with 1 corresponding to "I do not agree at all" and 10 to "I totally agree",** | | | | | | | | |
| **Do you consider that :** | | | | | | | | |
| 36 | My relatives are more likely than the general population to be infected. | | | | 1 ----------------------------------> 10 | | | |
| 37 | I'm having difficulty finding protective/hygiene equipment. | | | | 1 ----------------------------------> 10 | | | |
| 38 | I have confidence in the effectiveness of barrier measures. | | | | 1 ----------------------------------> 10 | | | |
| 39 | The equipment I have at my disposal is well suited for my work. | | | | 1 ----------------------------------> 10 | | | |
| 40 | I have set up my practice to comply with the barrier measures. | | | | 1 ----------------------------------> 10 | | | |
| 41 | I perform barrier measurements correctly. | | | | 1 ----------------------------------> 10 | | | |
| **Concerning your work rhythm, this last week :** | | | | | | | | |
| 42 | I have an inordinate amount of work. | | | | 1 ----------------------------------> 10 | | | |
| 43 | The time spent on the phone seems excessive to me. | | | | 1 ----------------------------------> 10 | | | |
| 44 | I receive conflicting instructions. | | | | 1 ----------------------------------> 10 | | | |
| 45 | I have some tough decisions to make. | | | | 1 ----------------------------------> 10 | | | |
| 46 | I am affected by the severity of some of my patients. | | | | 1 ----------------------------------> 10 | | | |
| 47 | I am affected by my patients’ anxiety or anguish. | | | | 1 ----------------------------------> 10 | | | |
| 48 | Reassuring my patients takes a lot of energy. | | | | 1 ----------------------------------> 10 | | | |
| 49 | I am worried about the follow-up of my chronic patients. | | | | 1 ----------------------------------> 10 | | | |
| **In this context of health crisis, this last week :** | | | | | | | | |
| 50 | I do quality work. | | | | 1 ----------------------------------> 10 | | | |
| 51 | My work makes sense to me. | | | | 1 ----------------------------------> 10 | | | |
| 52 | I feel in line with what I do in my job. | | | | 1 ----------------------------------> 10 | | | |
| 53 | My work is useful to the community. | | | | 1 ----------------------------------> 10 | | | |
| 54 | My remuneration is satisfactory. | | | | 1 ----------------------------------> 10 | | | |
| 55 | I am confident in the future / able to project myself in the coming weeks. | | | | 1 ----------------------------------> 10 | | | |
| 56 | When I get home, I can forget about my work. | | | | 1 ----------------------------------> 10 | | | |
| 57 | I feel like my work is taking up so much of my time that it’s impacting my personal life. | | | | 1 ----------------------------------> 10 | | | |
| 58 | I feel lonely in my work. | | | | 1 ----------------------------------> 10 | | | |
| 59 | I am thinking of using my right of withdrawal. | | | |  | | | |
| 60 | When I am concerned about certain situations, I have someone to talk to. | | | | 1 ----------------------------------> 10 | | | |
| 61 | I get the respect I deserve from my patients. | | | | 1 ----------------------------------> 10 | | | |
| 62 | I receive the support I need from my family and friends. | | | | 1 ----------------------------------> 10 | | | |
| **To address the current context :** | | | | | | | | |
| 63 | I feel competent enough to manage a COVID-19 patient. | | | | 1 ----------------------------------> 10 | | | |
| 64 | I feel overwhelmed by the amount and variety of information I receive. | | | | 1 ----------------------------------> 10 | | | |
| 65 | The information I receive answers to the questions I have. | | | | 1 ----------------------------------> 10 | | | |
| 66 | I know where to get the information I need. | | | | 1 ----------------------------------> 10 | | | |
| 67 | What are your three main sources of information? | | | | -General public information (television, radio...)  -Social networks for the general public  -Professional social networks  -Scientific Societies website  -Correspondent of the referring hospital  -Council of the Order  -Health Insurance Funds  -Santé Public France (Public Health France)  -DGS urgent  -Peer Group  -DMG  -Other, specify : | | | |
| 68 | If a coronavirus vaccine existed, would you vaccinate? | | | | Yes  No | | | |
| 69 | Do you have any other remarks to share with us? | | | | … | | | |
| This questionnaire has been completed. Your answers are saved.  Thanks for your participation! | | | | | | | | |
