# Supplementary material for: Family medicine practitioners’ stress during the COVID-19 pandemic: a cross-sectional survey
Source: BMC Fam Pract. 2021 Feb 14;22:36. doi: 10.1186/s12875-021-01382-3 (PMC7882249; doi:10.1186/s12875-021-01382-3)
Supplement: Supplementary file 2 — Additional file 2: Table S2. Topics classification of the open-ended question of the self-questionnaire. [file 12875_2021_1382_MOESM2_ESM.docx]

**Supplementary Table 2. Topics classification of the open-ended question of the self-questionnaire.**

| **Themes** | | **Number of**  **respondent** |
| --- | --- | --- |
| ***Themes addressed in the 31 positive assertions*** | | ***153*** |
| 1. Workload | | 24 |
|  | 1.1 Workload was lower than usual | 12 |
|  | 1.2 GPs had to reorganize their practice everyday | 16 |
| 2. Emotional requirements | | 40 |
|  | 2.1 Psychological toll | 22 |
|  | 2.2 Anxious about chronic patients not coming in consultation | 29 |
| 3. Conflict of values: not in line with the job | | 3 |
| 4. Feeling of economic insecurity | | 22 |
| 5. Working relationship and social report: Feeling alone, abandoned | | 9 |
| 6. Personal Protection Equipment (PPE) | | 38 |
|  | 6.1 Lack of PPE | 30 |
|  | 6.2 Difficulty for supplying PPE | 27 |
| 7. Access to the information | | 58 |
|  | 7.1 Too much information | 19 |
|  | 7.2 Contradictory, changing, unclear, hard to understand | 29 |
|  | 7.3 Incomplete information | 11 |
|  | 7.4 Too late, at the same time as general population | 8 |
| ***New themes addressed by GPs*** | | ***173*** |
| 1. New concepts about information and guidelines | | 36 |
|  | 1.1 Information by medias: a source to educate the population | 2 |
|  | 1.2 Information by medias: a source of anxiety | 7 |
|  | 1.3 Information by authorities: mistrust, loss of confidence. | 29 |
|  | 1.4 Difficulty in advising patients, as having a similar level of information. | 8 |
| 2. The place of GPs in the organization to the response to health crisis | | 72 |
|  | 2.1 Hospital-based organization | 43 |
|  | 2.2 Non-existent collaboration between GPs and hospital | 13 |
|  | 2.3 Improvement of the organization between GP themselves: a source of mutual help | 29 |
| 3. The feeling GPs about the health crisis | | 67 |
|  | 3.1 Useless, guilty | 7 |
|  | 3.2 Downgrade/Forgotten, left aside | 24 |
|  | 3.3 Angry for authorities | 25 |
|  | 3.4 GPs felt to be on the front line | 21 |
| 4. Other material elements | | 28 |
|  | 4.1 General population is not using the PPE well | 3 |
|  | 4.2 Diagnostic tests: lack of test, not reliable | 5 |
|  | 4.3 Treatment controversy: Hydroxychloroquine | 11 |
|  | 4.4 Vaccine controversy | 9 |
| 5. Personal life | | 13 |
|  | 5.1 Fear to transmit COVID-19 to the family | 8 |
|  | 5.2 Fear about themselves: pregnancy, chronic diseases… | 6 |
